# The Association of Family-Related Adversity With Fighting in Adolescents: Does Hopelessness Mediate This Association?

**DOI:** 10.3389/ijph.2021.607199

**Published:** 2021-03-29

**Authors:** Jaroslava Macková, Zuzana Dankulincova Veselska, Andrea Madarasova Geckova, Danielle Jansen, Jitse P. van Dijk, Sijmen A. Reijneveld

**Affiliations:** ^1^ Department of Health Psychology and Research Methodology, Faculty of Medicine, University of Pavol Jozef Šafárik, Kosice, Slovakia; ^2^ Graduate School Kosice Institute for Society and Health, PJ Safarik University, Kosice, Slovakia; ^3^ Department of Community and Occupational Health, University of Groningen, Groningen, Netherlands; ^4^ Olomouc University Social Health Institute (OUSHI), Olomouc, Czechia

**Keywords:** family-related adversities, fighting, hopelessness, adolescents, HBSC study

## Abstract

**Objectives:** To explore the association of family-related adversities with physical fighting, and whether this association is mediated by hopelessness.

**Methods:** The sample consisted of 3712 Slovak adolescents (mean age: 13.9, 50.7% girls). Participants answered questions regarding experienced family-related adversities, involvement in physical fighting in the last 12 months and the Hopelessness Questionnaire. First, the association of family adversities in general with fighting and of each of family-related adversity separately was assessed using linear regression models and second, mediation was assessed using the a*b product method with bootstrapped 95% confidence intervals

**Results:** Adolescents who had experienced at least one family adversity reported more frequent fighting. Similarly, each of reported family adversities (death of a parent, substance abuse problems of a parent, conflicts/physical fights, divorce) was associated with more frequent fighting among adolescents. The mediation effect of hopelessness was found in each association of family-related adversity with fighting.

**Conclusion:** These findings suggest that interventions to support adolescents who had experienced family adversities could among other things be directed at better coping with hopelessness.

## Introduction

The World report on violence and health (1) defines youth violence as an intentional use of physical force or power, threatened or actual against another person or group or community occurring among individuals aged 10–29 years, who are unrelated to each other, who may or may not know each other and these acts of violence usually take place outside of home. Examples of youth violence are bulling, physical fights, assault (with or without weapon) and gang violence. Fighting has been found to be associated with other risk behavior among adolescents such as drinking alcohol, smoking, drug use and missing school (2). Fighting among adolescents is a current public health problem in Slovakia, especially among boys. The most recent Health Behavior in School-aged Children Study (HBSC 2017/2018) in Slovakia revealed that 12% of boys and 5% of girls at the age of 15 years old were involved into physical fight at least three times in the last 12 months (3). This is a decrease in comparison to the previous HBSC study in 2013/2014 (4), where 19% of boys and 6% of girls reported involvement in physical fighting at least three times in the last 12 months. Findings from the last HBSC study (5) including 45 countries in Europe and Canada showed that on average 12% boys and 5% girls aged 15 years were involved into physical fights three or more times in the last 12 months. Adolescent fighting is thus a problem not only in Slovakia, but similarly in other countries. As with other types of behavior in adolescents, family adversities play a significant role in formation of violent behavior including fighting with effect being similar for boys and girls (6). Research has shown that adolescents from families where violence was present were more aggressive (7) and were more likely to be engaged in violent behavior (8–10). Not only violence, but also parental problem drinking (11) and living in a single-parent family (12) were associated with violent behavior in adolescents.

Several theories have been proposed to explain why violent behavior occurs more frequently in families that experience adversities. First, probably the most prominent theory how family factors contribute to violent behavior is the Social learning theory of Ref. 13 which explains how violent behavior is transmitted from parents to their offspring by observational learning as parents serve as role models of behavior for their children. However, observational learning of violent behavior may be considered as only one of the potential mechanisms linking family related adversities with fighting among adolescents. On top of violent behavior itself, emotional regulation can also be learned from parents. Therefore, we based for research on the more recent Tripartite Model of the Impact of the Family on Children’s Emotion Regulation and Adjustment (14). This model provides a broader context for how family factors affect adjustment in children, that combines the above. This model describes how family climate, parenting practices and parental characteristics (such as their own emotional regulation and mental health) affects emotional regulation of their offspring via observational learning. That emotional regulation in turn affects children’s adjustment (demonstrated by internalizing, externalizing problems, social competence etc.).

Hopelessness and its association with violent behavior, e.g., fighting among adolescents, has been previously found in the general population of adolescents (15, 16), but most evidence for this association comes from studies of adolescents living in impoverished suburban areas, public housing neighborhoods or inner-city neighborhoods (17–20). Much less attention has been devoted to how family adversities might create conditions where hopelessness occurs in adolescents. We expect that family adversities, similarly like neighborhood factors, can produce an environment where hopelessness is experienced and leads to violent behavior. Reference 21 found that chronic, uncontrollable stressors are associated with experiencing hopelessness in adolescents. Family adversities are also events that produce chronic and uncontrollable stressors (22), which have been shown to be associated with experienced hopelessness (23, 24). Moreover, Ref. 18 postulated that the process of abandoning hope implies that adolescents are more likely to engage in violent behavior if they believe that failure is an inevitable part of their future. We expect that this process might occur when adolescents are experiencing serious family adversities as these are usually out of their control. As evidence on hopelessness as a mechanism connecting family adversities with fighting is lacking, this issue requires further study.

Therefore, the aim of the study was first to examine the association of family-related adversity with fighting in adolescents generally and separately for each type of family-related adversity, and second to assess if the experienced hopelessness in adolescents mediates these associations. We expected that experienced family-related adversity is associated with more frequent fighting in adolescents. Second, we expected that hopelessness mediates these associations.

## Methods

### Sample and Procedure

We used data from the Health Behavior in School-aged Children (HBSC) study that was conducted in 2018 in Slovakia. In order to obtain a representative sample, we used a two-step sampling procedure. First, 140 larger and smaller schools located in rural as well as urban areas from all regions of Slovakia were randomly selected from a list of all eligible schools in Slovakia and asked to participate in the study. The response rate (RR) of schools was 77.9%. Second, we obtained data from 8,405 adolescents aged 11–15 years old (RR = 77.9%; mean age = 13.43; 50.9% boys) by using online self-report questionnaires. The questionnaire on hopelessness was filled in only by 13- and 15-year old pupils; therefore, we excluded respondents younger than 13 (*N* = 3716). After the exclusion of respondents with missing answers (*N* = 977), a final sample of 3712 Slovak adolescents remained (mean age: 13.9, 50.7% girls).

Our study was approved by the Ethics Committee of the Medical Faculty at P.J. Safarik University in Kosice (16N/2017). We informed parents about the study via the school administration, and they could opt out if they disagreed with their child’s participation. Participation in the study was fully voluntary and confidential with no explicit incentives provided for participation.

### Measures

*Family-related adversity* regarded various negative events in the family, as measured by the following questions: “Have you ever experienced any of the following events?: 1) Problems with alcohol or drugs of one of your parents? 2) Serious conflicts or physical fights between your parents? 3) Divorce of your parents? 4) Death of your mother or father?” Questions could be answered Yes or No. The questions were derived from the International Self-Report Delinquency Study 2: Standard Student Questionnaire (25) and was used previously in a sample of Slovak adolescents (26). We used this variable in analysis in two ways. First, a positive answer (yes) on at least one question from the four questions was classified as a family-related adversity, whereas instances in which all answers were negative were classified as no family-related adversity. This allows us to create a dichotomous variable “Family-related adversity” (at least one family-related adversity vs. no family adversity). Second, we used separately each question on family-related adversity as dichotomous variable (specific adversity is present vs. specific adversity is not present).

*Fighting* was measured by the question: “During the past 12 months, how many times were you in a physical fight?” This question was derived from the Youth Risk Behavior Survey Questionnaire (27), with the following possible answers: “I have not been in a physical fight in the past 12 months” (0), “One time” (1), “Two times” (2), “Three times” (3) and “Four or more (4)”.

*Hopelessness* was measured using the Hopelessness Questionnaire (28), which consists of five items (item example: “I might as well give up because I can’t make things better for myself”), with answer categories yes/no. Then the sum score was calculated of the total number of “yes” responses; a higher score indicates that more hopelessness is experienced. Cronbach’s alpha for the total scale in this sample was 0.79.

*Perceived socioeconomic position status of the family (SEP)* was measured as a possible confounder by the Perceived family wealth scale (29), which measures adolescents’ perception of their own family’s socioeconomic circumstances. Responses were: “not at all well off” (0), “not so well off” (1), “average” (2), “quite well off” (3), “very well off” (4). A higher score indicates a higher perceived SEP of the family.

### Statistical Analyses

First, we described the background characteristics of the sample using descriptive statistics. We described differences between girls and boys, older and younger adolescents and those with lower and higher SEP in experiencing family-related adversities, fighting and hopelessness using Chi-square statistics, t-test for independent samples and one-way ANOVA. Next, we assessed the associations of family-related adversity, hopelessness and fighting using multivariate linear regression models adjusted for gender, age and SEP. Third, we assessed the association of each family adversity (death of parent, substance abuse problems of parent, conflicts or physical fights between parents, divorce of parents), hopelessness and fighting using multivariate linear regression models adjusted for gender, age and SEP. All regression analyses were performed on 5,000 bootstrap samples. Fourth, we explored whether hopelessness mediated the association of family-related adversity with fighting. Finally, we ran same analysis of mediation effect of hopelessness in the association of each of family adversities (death of parent, substance abuse problems of parent, conflicts or physical fights between parents, divorce of parents) with fighting. The mediation analysis was performed using the PROCESS macro model 4 (30) and was controlled for gender, age and SEP and was performed on 5,000 bootstrap samples. The indirect effect was calculated using the a*b product method and bootstrapped 95%-confidence intervals (CI) were calculated. All analyses were performed in SPSS v. 23 (IBM Corporation, New York, NY, USA) for Windows.

## Results

### Description of the Sample

Table 1 shows the descriptive statistics of the study sample regarding gender, age, SEP, family-related adversity, hopelessness and fighting. It shows that girls–compared to boys—reported experiencing at least one family-related adversity more frequently, and this also applied to substance abuse problems of parent, conflicts or physical fights between parents and divorce of parents. Boys were involved in physical fights more frequently and girls experienced higher levels of hopelessness than boys. Older adolescents reported more frequently than younger ones that they experienced at least one family-related adversity, substance abuse problems of parent and conflicts or physical fights between parents. Adolescents with a lower SEP reported more frequently than adolescents with higher SEP that they experienced at least one but also every specific family-related adversity except death of a parent. Moreover, low SEP adolescents also reported higher level of hopelessness.

**TABLE 1:** Background characteristics of the sample, overall and by gender, age and socioeconomic position (3,712 Slovak adolescents aged 13–15, collected in 2018).

			Gender			Age				Socioeconomic position		
		N (%)	Boys	Girls	*p*-value	13-year	14-year	15-year	*p*-value	Higher	Lower	*p*-value
Gender												
Girls		1,882 (50.7)	—	—	—	669 (51.3)	732 (51.0)	481 (49.5)		393 (46.0)	1489 (52.1)	
Boys		1,830 (49.3)	—	—	—	636 (48.7)	704 (49.0)	490 (50.5)	ns	461 (54.0)	1369 (47.9)	**
Family-related adversity^1^												
Any adversity		1,256 (33.8)	550 (30.1)	706 (37.5)	***	393 (30.1)	498 (34.7)	365 (37.6)	**	220 (25.8)	1036 (36.2)	***
Specific family adversities												
Death of parent		131 (3.5)	64 (3.5)	67 (3.6)	ns	43 (3.3)	46 (3.2)	42 (4.3)	ns	29 (3.4)	102 (3.6)	ns
Substance abuse problems of parent		409 (11.0)	176 (9.6)	233 (12.4)	**	122 (9.3)	162 (11.3)	125 (12.9)	*	51 (6.0)	358 (12.5)	***
Conflicts or physical fights between parents		603 (16.2)	223 (12.2)	380 (20.2)	***	186 (14.3)	223 (15.5)	194 (20.0)	**	101 (11.8)	502 (17.6)	***
Divorce of parents		798 (21.5)	356 (19.5)	442 (23.5)	**	264 (20.2)	322 (22.4)	212 (21.8)	ns	148 (17.3)	650 (22.7)	**
	Range	Mean (SD)										
Age	13–15	13.90 (0.8)	13.9 (0.8)	13.9 (0.8)	ns	—	—	—	—	14.0 (0.8)	13.9 (0.8)	***
Socioeconomic position^2^	0–4	2.87 (0.8)	2.9 (0.8)	2.8 (0.8)	*	2.9 (0.9)	2.9 (0.8)	2.8 (0.8)	**	—	—	—
Fighting^3^	0–4	0.61 (1.1)	0.8 (1.3)	0.4 (0.9)	***	0.7 (1.2)	0.6 (1.1)	0.56 (1.1)	ns	0.6 (1.1)	0.6 (1.1)	ns
Hopelessness^4^	0–5	0.85 (1.4)	0.8 (1.3)	0.9 (1.5)	***	0.9 (1.4)	0.8 (1.4)	0.8 (1.3)	ns	0.7 (1.3)	0.9 (1.4)	**

*p*-values are based on chi-square tests for categorical variables and on *t*-tests for continuous variables when comparing two groups and One-way ANOVA when comparing three groups; ^*^ *p* = 0.05, ^**^ *p* = 0.01, ^***^ *p* ≤ 0.001.

^1^ Experiencing any of family adversities.

^2^ A higher score indicates a higher socioeconomic status of family.

^3^ A higher score indicated more frequent fighting.

^4^ A higher score indicated higher level of hopelessness.

### The association of family-related adversity, hopelessness and fighting

Table 2 presents the association of family-related adversity, hopelessness and fighting. We found that adolescents who experienced at least one adversity in the family reported more frequent fighting during the last 12 months. In addition, adolescents who reported at least one of the family adversities experienced a higher level of hopelessness. Moreover, adolescents who experienced a higher level of hopelessness also reported more frequent fighting in the last year. We noticed that after adding hopelessness to the model, the association of family-related adversity with fighting weakened, and therefore, we expected a mediating effect of hopelessness and examined it in the next step. R-square of this model was 0.06.

**TABLE 2:** The association of family related adversity and hopelessness with fighting adjusted for gender, age and perceived socioeconomic status: results from multivariate linear regression models leading to regression coefficients (B) and 95% confidence intervals (CI) (3,712 Slovak adolescents aged 13–15, collected in 2018).

	B (95%CI) (adjusted for gender, age and SEP)	B (95%CI) (adjusted for gender, age and SEP)
Family-related adversity	0.21 (0.13; 0.29)***	0.17 (0.10; 0.25)***
Hopelessness	—	0.09 (0.07; 0.12)***
R-square^1^		0.06***

SEP—perceived socioeconomic status of the family; ^*^ *p* < 0.05, ^**^ *p* < 0.01, ^***^ *p* < 0.001.

^1^ Model includes age, gender, SEP, family-related adversity and hopelessness as independent variables.

Table 3 presents the association of each separate family adversity, hopelessness and fighting. Out of the explored family adversities, death of a parent was most strongly associated with fighting. Substance abuse problems of parents and conflicts or physical fights between parents were also connected with fighting among adolescents. The weakest association was found between divorce of parents and fighting. We also noticed in all these models that adding hopelessness weakened the associations of the separate family adversities with fighting, and therefore, we decided to explore its role as mediator in the next step.

**TABLE 3:** The association of each of family-related adversities (death of parents, substance abuse problems of parents, conflicts or physical fights between parents, and divorce of parents) and hopelessness with fighting adjusted for gender, age and perceived socioeconomic status: results from multivariate linear regression models leading to regression coefficients (B) and 95% confidence intervals (CI) (3,712 Slovak adolescents aged 13–15, collected in 2018).

	Fighting (adjusted for gender, age and SEP) B (95% CI)	Fighting (adjusted for gender, age and SEP) B (95% CI)
Death of parent Hopelessness	0.35 (0.14; 0.57)** —	0.30 (0.11; 0.49)** 0.10 (0.07; 0.13)***
Substance abuse problems of parents Hopelessness	0.30 (0.18; 0.43)*** —	0.24 (0.12; 0.37)*** 0.09 (0.07; 0.12)***
Conflicts or physical fights between parents Hopelessness	0.26 (0.15; 0.37)*** —	0.20 (0.10; 0.32)*** 0.09 (0.07; 0.12)***
Divorce of parents Hopelessness	0.13 (0.04; 0.22)** —	0.11 (0.02; 0.20)* 0.10 (0.07; 0.13)***

SEP—perceived socioeconomic status of the family; ^*^ *p* < 0.05, ^**^ *p* < 0.01, ^***^ *p* < 0.001.

### The mediation effect of experienced hopelessness

**FIGURE 1:**
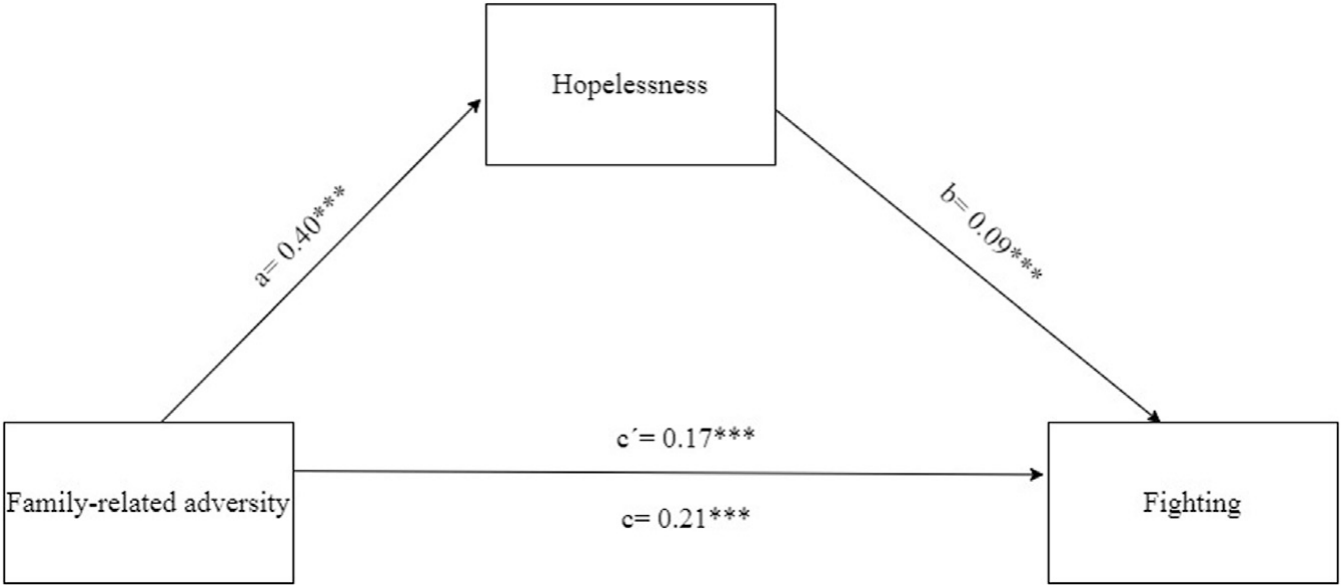
The mediation effect of hopelessness in the association of family-related adversity and fighting adjusted for gender, age and SEP (4,274 Slovak adolescents, age 13–15, collected in 2018, HBSC study).

We assessed the mediation effect of hopelessness in the association of family-related adversity with fighting. Results from mediation analysis (Figure 1) suggested that experienced hopelessness mediated the association of family related adversity with fighting, with the indirect effect of *ab* = 0.04 (95%-CI: 0.02–0.05).

**FIGURE 2:**
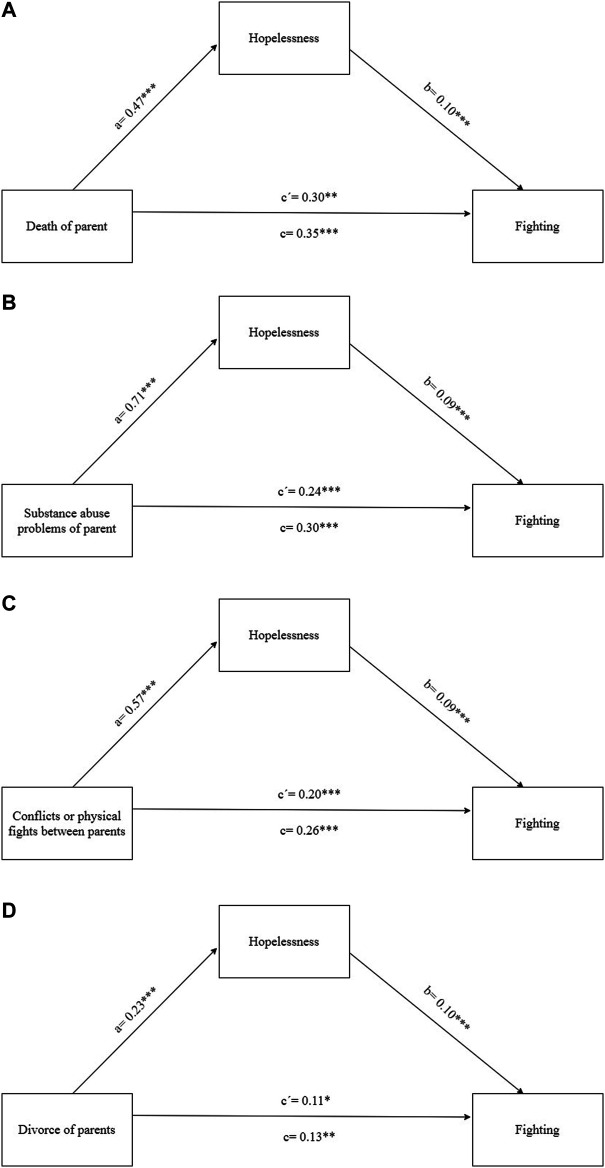
**(A)** The mediation effect of hopelessness in the association of death od parent and fighting adjusted for gender, age and SEP (4,274 Slovak adolescents, age 13–15, collected in 2018, HBSC study (Health Behaviour of School-aged Children, Slovakia, 2018)). **(B)** The mediation effect of hopelessness in the association of substance abuse problems of parent and fighting adjusted for gender, age and SEP (4274 Slovak adolescents, age 13–15, collected in 2018, HBSC study). **(C)** The mediation effect of hopelessness in the association of conflicts or physical fights between parents and fighting adjusted for gender, age and SEP (4,274 Slovak adolescents, age 13–15, collected in 2018, HBSC study). **(D)** The mediation effect of hopelessness in the association of divorce between parents and fighting adjusted for gender, age and SEP (4,274 Slovak adolescents, age 13–15, collected in 2018, HBSC study).

Next, we assessed the mediation effect of hopelessness in the association of the separate family-related adversities with fighting. We found a mediation effect in association of each of family-related adversities with fighting. These mediation effects (*ab*) were 0.05 (95% CI: 0.02–0.08) in the association of death of a parent with fighting; *ab* = 0.07 (95% CI: 0.04–0.10) in the association of substance abuse problems of parent with fighting; *ab* = 0.05 (95% CI: 0.03–0.08) in the association of conflicts or physical fights between parents with fighting and *ab* = 0.02 (95% CI: 0.01–0.04) in the association of divorce with fighting. These results show that the strongest association was found between substance abuse problems of parent and hopelessness (B = 0.71***) and the mediation effect of hopelessness in the association of substance abuse problems with fighting was the biggest. Out of the explored family-related adversities, divorce of parents was the most weakly associated with hopelessness among adolescents (B = 0.23***) and similarly we found the smallest mediation effect of hopelessness in the association of divorce with physical fights (Figure 2A-D).

## Discussion

The aim of this study was to assess whether family-related adversity is related to fighting among adolescents and whether this association is mediated by hopelessness.

We found that adolescents who experienced any family adversity were more frequently involved in fighting. This finding is in line with previous research (7, 8, 10–12). The least surprising finding is, that adolescents who experienced serious conflicts and physical fights between parents, were more frequently involved in fighting than adolescents who did not have such experience. We can explain this finding using the term “intergenerational transmission of violence” (e.g., Ref. 31; 35), which describes how violence present in the family is “transmitted” to the children, and they may later also become participants in violent behavior. This intergenerational transmission can be explained such that various types of behaviors, violent behaviors included, are discovered by vicarious learning, which means that witnessing family violence is enough for this behavior to be repeated later in their life (13, 32). This mechanism is best described in the Social learning theory of Ref. 13; which explains how children learn various types of behavior from their role models and parents are one of these.

Not only violence in the family, but also other adverse experiences in the family were associated with more frequent fighting among adolescents. We found that substance abuse problems of parents were connected to fighting, which is in line with previous research (11, 33). A broader context for interpretation is provided by the Tripartite Model of the Impact of the Family on Children’s Emotion Regulation and Adjustment (14). This model describes how the emotional regulation of children is affected by their personal characteristic, characteristics of their parents (including their own reactivity and emotional regulation, mental health, family history), the emotional climate of the family, parenting practices and how this emotional regulation is associated with children’s adjustment. We can expect difficulties in parental emotional regulation in cases when they abuse alcohol (see Ref. 34, and these insufficient coping strategies may be transferred to the children by observational learning. Moreover, research has shown that the development of impulse control circuits differs between children of parents who abuse alcohol or other drugs [44] and other children. This may also contribute to aggressive behavior (35). Moreover, several studies (for review see Ref. 36) on the development of children of alcoholics (COA) showed that adolescents from these family are more likely to develop internal (such as depression and anxiety) or external problems (substance abuse, conduct problems).

The adolescents who experienced the death of a parent were also more frequently involved in physical fights. Previous studies on children bereavement process after losing a parent brought mixed results (for review see ref. 37) but highlighted that moderating factors such as the family mourning process and family functioning may play a very important role. These factors have not been included in our study, but our previous research showed that family adversities including death of a parent are associated with worse family functioning (38). Parenting practices and emotional climate of the family are affected by parental characteristics (such their own ability to express and regulate emotions), and these factors affect the emotional regulation of adolescents. Adolescents learn from their parents via observational learning how to express, regulate and cope with emotions and this might result in problematic adjustment (depending on emotional regulation of parents which serves as a model), which is demonstrated by violent behavior. These mechanisms have been hypothesised by The Tripartite Model of the Impact of the Family on Children’s Emotion Regulation and Adjustment (14).

Out of the explored family adversities, divorce had the weakest association with fighting among adolescents. Previous research of Ref. 39 showed that adolescents who experienced divorce of parents were at greater risk to suffer from externalizing problems, including delinquent and aggressive behavior. They found that this association weakened in time. We did not include the time factor in our study, i.e., we did not ask adolescents how long it has been since the divorce of parents. The resulting varying length may have contributed to the weak association of these variables that we found. Second, similarly as in the case of death of parent, there are other variables that might affect this association, e.g., presence of non-residential parent and quality of relationship between him or her and adolescent (e.g., Ref. 40).

Next, we found that the association between family-related adversity and fighting is mediated by experienced hopelessness in adolescents. To our knowledge, no previous study has examined this pathway, but support for this finding can be found in studies assessing the components of this mediation in parts, i.e., the association of family adversities with hopelessness (23) and the association of hopelessness with violent behavior (e.g., Refs 15, 17, 20, and 41). An explanation for this finding could be found in Seligman’s Theory of Learned Helplessness (42), i.e., that an uncontrollable trauma produces a sense of powerless and an inability to control or change the situation. Reference 43 identified hopelessness as an outcome of learned helplessness. Moreover, Ref. 20 state that “the roots of hopelessness are formed when emotional needs are not met during early childhood”, which can explain why family adversities create an environment in which hopelessness might occur. The process of abandonment of hope (18) states that adolescents are more likely to engage in violent behavior if they believe that failure is an inevitable part of their future. This belief is a crucial part of hopelessness; therefore, it is comprehensible that adolescents who are hopeless are fighting more frequently. Our study adds to this that not only factors in the neighborhood where adolescents are living, but also familial factors, such as family adversities, may create an environment in which hopelessness occurs and may result in violent behavior.

### Strengths and limitations

The main strength of our study is that we used a national representative sample of adolescents; therefore, our findings can be generalized to the whole population of Slovak adolescents aged 13–15 years old. However, some limitations should also be mentioned. First, we used self-reported questionnaires, meaning that some participants might not be completely honest in their answers. Second, the cross-sectional design of this study does not allow us to make conclusions about causality in the examined relations. Third, the fighting variable was not continuous, and therefore it was not normally distributed, but bootstrapping might have solved this issue, as it does not expect normal distribution of data. A potential limitation is that the R-square of the model was quite small, though to be expected given the independent variables were dichotomous. However, smaller R-square might still invites for assessing other explanatory factors.

### Implications

We found family-related adversity to be associated with physical fighting, with hopelessness mediating this association, which has implications for practice and future research. First, we need interventions aimed at preventing violent behavior, especially for adolescents from families experiencing adversities. Moreover, these adolescents experienced higher level of hopelessness, and it would be helpful to pay attention to strategies for coping with feelings of hopelessness. All of the above may be useful for care providers who work with young people—psychologists, psychotherapists, youth workers, teachers etc. Future research may benefit from longitudinal studies to explore the causal relation between family-related adversity and fighting in adolescents. A longitudinal study might also reveal how the process of abandoned hope works and what factors might reverse it. Moreover, next studies might pay attention to other family-related factors such as family structure.

### Conclusion

Adolescents who experienced family-adversity were more frequently involved in physical fighting. This association was mediated by experienced hopelessness in adolescents. These findings suggest that youth workers working with adolescents who have experienced family adversities should help them cope with hopelessness.

## Data Availability

The raw data supporting the conclusions of this article will be made available by the authors, without undue reservation.
